# Infertility and Hematologic Malignancies in Low-Resource Settings: Challenges and Perspectives

**DOI:** 10.7759/cureus.103959

**Published:** 2026-02-20

**Authors:** Monsif Fadi, Chaimaa Hilali, Fatima Azzahra Lahlou, Nouama Bouanani

**Affiliations:** 1 Oncopathology, Center for Doctoral Studies (CEDoc) Cancer Biology and Environment Laboratory, Mohammed VI University of Health Sciences (UM6SS), Casablanca, MAR; 2 Biochemistry, Faculty of Medicine, Mohammed VI University of Health Sciences (UM6SS), Casablanca, MAR; 3 Biochemistry, Health and Environment Laboratory, Ain Chock Faculty of Sciences, Hassan II University, Casablanca, MAR; 4 Hematology, Faculty of Medicine, Mohammed VI University of Health Sciences (UM6SS), Casablanca, MAR

**Keywords:** global epidemiology, hematologic malignancies (hm), infertility, public health care, traitement

## Abstract

Infertility represents a significant challenge in reproductive health, with implications extending beyond individual well-being to public health systems worldwide. Despite its high prevalence, fertility impairment remains insufficiently integrated into cancer survivorship care, particularly in settings with limited healthcare resources. Hematologic malignancies constitute a distinct clinical context in which infertility risk is especially relevant, as these diseases often affect adolescents and young adults and are increasingly associated with long-term survival. However, standard treatments such as high-dose chemotherapy, radiotherapy, and hematopoietic stem cell transplantation carry a substantial risk of gonadal toxicity and long-term reproductive impairment. More recently, immunotherapy and targeted therapies have emerged as effective therapeutic options, yet their long-term effects on fertility remain poorly characterized and warrant further investigation. Although effective fertility preservation strategies are available, including sperm cryopreservation, oocyte vitrification, and embryo freezing, access to these interventions remains limited due to financial, structural, and logistical barriers, as well as insufficient clinician awareness. Integrating fertility preservation into routine hematologic care through enhanced education, institutional protocols, and supportive health policies is essential to address this under-recognized complication and improve long-term quality of life for cancer survivors in resource-limited settings.

## Editorial

Infertility is a major global public health issue that affects a substantial proportion of individuals of reproductive age, yet it remains insufficiently addressed in cancer survivorship care. Epidemiological data indicate that infertility affects a significant proportion of couples worldwide, with marked regional disparities reported across low- and middle-income countries. Large population-based analyses have consistently shown higher prevalence estimates in sub-Saharan Africa compared with high-income regions [1].

Hematologic malignancies represent a unique clinical context in which infertility risk is especially relevant, as these diseases frequently affect adolescents and young adults and are increasingly associated with long-term survival. However, the therapeutic strategies required to achieve disease control, including high-dose chemotherapy, radiotherapy, and hematopoietic stem cell transplantation (HSCT), are known to induce severe gonadal toxicity. A recent meta-analysis demonstrated that more than 30% of patients undergoing HSCT develop permanent infertility, with higher rates observed among female survivors. These findings confirm that fertility impairment is not a rare complication but rather a common and predictable consequence of modern hematologic treatments [2,3].

Despite the growing body of evidence, infertility remains a largely underestimated complication in routine hematology practice. Fertility preservation strategies such as sperm cryopreservation, oocyte vitrification, and embryo freezing are now well established and recommended by international guidelines prior to initiating gonadotoxic therapies. Several clinical reviews have emphasized that early fertility counseling significantly improves uptake of preservation methods and patient satisfaction. Nevertheless, in real-world practice, fertility discussions are frequently delayed or omitted, particularly in low-resource environments where survival remains the primary clinical focus [4].

The epidemiological burden of infertility in low-resource settings further reinforces the need for integrated oncofertility care. While precise cancer registry data are often lacking, regional infertility studies consistently report high prevalence rates, reflecting both biological and health system factors. In sub-Saharan Africa, for example, infertility prevalence is among the highest globally, with important psychosocial consequences including stigma, marital instability, and psychological distress. When combined with increasing cancer survival, these data suggest that infertility will represent a growing survivorship challenge in hematologic populations [1].

Structural and logistical barriers play a central role in limiting access to fertility preservation in low-resource settings. These include the absence of cryopreservation facilities, lack of trained reproductive specialists, financial constraints, and limited clinician awareness of oncofertility principles. Even in tertiary centers, fertility preservation is often unavailable or financially inaccessible. Recent reviews in oncofertility highlight that inequities in access remain a major ethical and clinical concern, particularly for patients in low-resource healthcare systems [5].

In low-resource settings, pragmatic strategies such as developing simplified fertility counseling protocols, establishing referral networks to regional reproductive centers, and partially funding basic cryopreservation services through public or institutional funding could significantly improve access to fertility preservation. Telemedicine-based counseling and clinician training programs also represent cost-effective solutions to reduce inequities in oncofertility care.

In our institution, we conducted a local awareness survey among hematology and oncology practitioners to assess current practices regarding fertility preservation. However, most physicians acknowledged infertility as an important survivorship issue; fewer than 40% reported systematically discussing fertility risks before treatment initiation. Furthermore, less than 20% had access to dedicated fertility preservation services, and the majority of patients reported that they had not received adequate information about reproductive options. These findings mirror international observations and confirm that significant gaps persist between guidelines and clinical practice.

In conclusion, infertility is a common and clinically significant complication of hematologic malignancies that remains insufficiently addressed in low-resource settings. Integrating fertility preservation into standard cancer care requires not only technical resources but also clinician education, institutional protocols, and national health policies. Recognizing reproductive health as a fundamental component of cancer survivorship is essential to improving long-term quality of life for patients with hematologic malignancies.
